# Fairness as an afterthought: An American perspective on fairness in model developer-clinician user collaborations

**DOI:** 10.1371/journal.pdig.0000386

**Published:** 2023-11-20

**Authors:** John Banja, Judy Wawira Gichoya, Nicole Martinez-Martin, Lance A. Waller, Gari D. Clifford

**Affiliations:** 1 Center for Ethics, Emory University, Atlanta, Georgia, United States of America; 2 Department of Radiology and Imaging Sciences, Emory University School of Medicine, Atlanta, Georgia, United States of America; 3 Stanford University Center for Biomedical Ethics, Stanford University, Stanford, California, United States of America; 4 Department of Biostatistics and Bioinformatics, Rollins School of Public Health, Emory University, Atlanta, Georgia, United States of America; 5 Department of Biomedical Informatics, Emory University School of Medicine, Atlanta, Georgia, United States of America; 6 Department of Biomedical Engineering, Georgia Institute of Technology, Atlanta, Georgia, United States of America; Technical University Munich, GERMANY

## Abstract

Numerous ethics guidelines have been handed down over the last few years on the ethical applications of machine learning models. Virtually every one of them mentions the importance of “fairness” in the development and use of these models. Unfortunately, though, these ethics documents omit providing a consensually adopted definition or characterization of fairness. As one group of authors observed, these documents treat fairness as an “afterthought” whose importance is undeniable but whose essence seems strikingly elusive. In this essay, which offers a distinctly American treatment of “fairness,” we comment on a number of fairness formulations and on qualitative or statistical methods that have been encouraged to achieve fairness. We argue that none of them, at least from an American moral perspective, provides a one-size-fits-all definition of or methodology for securing fairness that could inform or standardize fairness over the universe of use cases witnessing machine learning applications. Instead, we argue that because fairness comprehensions and applications reflect a vast range of use contexts, model developers and clinician users will need to engage in thoughtful collaborations that examine how fairness should be conceived and operationalized in the use case at issue. Part II of this paper illustrates key moments in these collaborations, especially when inter and intra disagreement occurs among model developer and clinician user groups over whether a model is fair or unfair. We conclude by noting that these collaborations will likely occur over the lifetime of a model if its claim to fairness is to advance beyond “afterthought” status.

## Author summary

This essay has two parts. The first part explains why a universal, all-inclusive definition of fairness that could ethically inform, justify, and standardize the ways machine learning models operationalize fairness has not emerged, at least in the United States. This explains to some degree why prominent healthcare groups that have offered ethical guidelines or recommendations for machine learning development seem to treat fairness as vitally important yet gloss over attempts to define it. The second part of this essay traces the implications of a failure to adopt a one-size-fits-all definition and how that failure can affect the moral contours of the model developer-clinician user relationship. The importance of this conversation is heightened by the fact that machine learning models are virtually unregulated in the United States outside of general safety considerations; no methodological framework for identifying fairness-related issues and incorporating mitigation techniques in machine learning design exists; model developers might not be particularly sensitive towards considering how fairness plays out in their model; and “honest” disagreement can exist between model developers and clinician users over whether a given model is fair or unfair. We conclude by noting that if achieving algorithmic “fairness” is as challenging as we believe it to be, then 1) conceptualizations of fairness will be highly dependent on the specific use case under scrutiny for their content, 2) model developers and clinician users will need to be keenly sensitive as to how fairness impacts patient populations in those cases, and 3) model developers, clinician users, and the populations impacted by the model will need to engage in collaborative efforts throughout the life of their models that aim at operationalizing and realizing justifiable comprehensions and applications of fairness practices.

## Introduction

In a 2021 essay, Judy Gichoya and her co-authors noted that although virtually every contemporary code or set of ethical guidelines on the implementation of machine learning technologies mentions the moral importance of fairness, “(their) engagement with fairness tends to be either minimal or vague…(they) lack discussion of fairness or the relationship between these (machine learning) tools and the broader health equity context.”[1] The authors refer to how various positional statements [2–5] treat fairness as an “afterthought” whose conceptualization or definition is “often conspicuously absent or ineffectually vague.”[1] Other scholars have echoed Gichoya et al’s frustration, such as Luke Munn, who condemned ethical guidelines for artificial intelligence as “meaningless” and “toothless” [6].

This essay responds to these criticisms by first explaining why fairness, at least from an American perspective, is indeed an elusive concept to define and operationalize, not to mention regulate, in machine learning (ML) development. There are currently no regulations in the U.S. that specify fairness in ML construction or how fair models are to be designed [7]. While courts can penalize individuals or corporations for violating stipulations of the U.S. Civil Rights Act [8], and the Federal Trade Commission (FTC) can penalize corporations for using a model that frankly discriminates [9], that legal scrutiny has not yet been articulated in the form of algorithmic fairness requirements, as it has in the European Union vis a vis the General Data Protection and Regulation Act [10–12].

The second half of the paper describes the effects of this absent or omitted definition of fairness on clinician users when they engage ML designers or developers. In this section, we also include a discussion about the “moral agency” or “the ethics of being a model developer,” about which there is considerable moral worry but a paucity of research. The essay concludes with a series of considerations bearing on how model developers and clinician users might collaborate and align their comprehensions of fairness as those models are deployed among patient populations.

This essay will therefore be unlike many, perhaps most, contemporary papers on ML fairness that focus on technical or computational adjustments to an existing model’s data, its collection and curation, choice of model, etc., that make it “more fair.” These papers typically assume or propose a definition of fairness, e.g., predictive parity, distributional equality, or equalized odds, and then suggest how a model might be mathematically adjusted to accommodate the definition [13–14]. This paper will instead discuss the philosophical underpinnings and subsequent quandaries that background model development and the various social justice considerations they can present to model developers and clinician users. Ultimately, we hope this essay supplies some ethical “forethought” to the abbreviated “afterthought” status of fairness in ML models so that its conversation and related debates lead to more enlightened, transparent, and democratic conceptions of algorithmic fairness.

## Part I

### The elusiveness of a standard definition of fairness in ML models

Moral concerns over algorithmic fairness originate from a much-chronicled history of socioeconomic disparities and inequalities that certain populations in the West have experienced for centuries. As the interest of this journal is health care, we center comprehensions of unfairness on the phenomenon of health inequities, i.e., unjust (and, therefore, legally and ethically unacceptable) practices in health care distributional space that prevent or discourage the availability of desirable health care services to historically marginalized subgroups [15]. Health inequities do not only conjure up health inequalities. They especially denote, because they are caused by, historically evolved biases or prejudices that unjustly favor one individual or group over another [16–17]. Models that therefore rely on variables or computational formulae that sustain such inequities are patently unfair and, therefore, unjust.

Space only allows a brief comment on why contemporary approaches towards developing a useful and operational conception of fairness are complicated by philosophical, political, and socioeconomic disagreements. Nevertheless, these philosophical and political controversies over fairness explain, or so we believe, its “afterthought” status in the ethical guidelines ML literature, especially as it might occur among American scholars but also among data scientists throughout the West.

We begin by examining perhaps the most common strategy for achieving fairness in the literature. Called “fairness through unawareness,” this strategy seeks to eliminate prejudices or biases from datasets that unfairly disadvantage certain subpopulations at risk for discrimination [18–20]. A good example is the August 2022 U.S. Department of Health and Human Services (DHHS) proposal to extend Section 1557 of the Affordable Care Act’s antidiscrimination requirements to clinical algorithms if the algorithm’s outputs result in discrimination from the use of race or ethnicity variables [21]. This concern about models using protected or sensitive variables in discriminatory ways is pre-eminent among bias mitigation sensibilities and plays a central role in the FTC’s understanding of fairness [21,22].

A major problem with this strategy, however, is that a protected or sensitive variable can have significant predictive value in certain clinical contexts, such as age in sepsis prediction models [23] or race in cancer screening practices [24]. It could therefore be clinically counter-productive to purge certain models of protected attributes if they could dramatically improve the model’s predictive accuracy or the assignment of treatment options (thus prompting the reverse notion of “fairness through awareness” [20]). Furthermore, deleting a protected variable to advance the justice interests of one group might cause a deterioration in predictive accuracy for another group and therefore preserve allegations of discrimination (albeit now against a majority group!) [18,22]. Yet, another problem with a blanket removal of protected attributes is that certain models can nevertheless “infer” the protected attribute, even if it is eliminated, and continue to maintain its discriminatory impact [25,26]. Consequently, “fairness through unawareness” is not always effective, possible, or even ethically warranted.

A second conceptualization of fairness proposes not only equal predictive accuracy across groups but also equalized *error rates* across groups [13,18]. This fairness intuition—called “fairness as equalized odds”—was a prominent criticism of the much-discussed COMPAS recidivism prediction model inquiry reported by ProPublica in 2016 [27]. COMPAS is a widely used model in the U.S. judicial system that provides courts with likely-to-reoffend scores for individuals coming up for parole or who are coming up for trial. Receiving a high COMPAS score among the former means they will be denied parole and spend longer time in jail. Receiving a high score among the latter means they will be denied bond and remain in jail until their case comes up for trial [28].

What captured the interest of social justice scholars when the ProPublica report appeared was that the model’s true positive or likely-to-reoffend scores were equally accurate between white and Black individuals. However, its false positive scores, i.e., likely-to-reoffend predictions that were wrong, heavily disadvantaged Black individuals by way of their having to spend more jail time. Alternatively, its u*nlikely*-to-reoffend (or false negative) ratings heavily advantaged white persons by way of their being awarded bond or parole [27,29,30]. Remarkably and as data scientists proceeded to demonstrate, it might be mathematically impossible to satisfy both fairness goals simultaneously—in the COMPAS case, a nondiscriminatory accuracy score *plus* a nondiscriminatory impact rating [31,32]. As discussed in Part II, this implies that clinician users and model developers might need to collaborate and agree on which of their comprehensions of fairness will be algorithmically overriding (e.g., predictive accuracy versus group error rates) and/or how to make certain adjustments in the model that satisfy their mutually negotiated vision of fairness.

This suggests a third and all-inclusive problem with fairness comprehensions. Developers of models that are presumably “fair” should be held accountable for their deliverables, and they therefore should be willing to open their models and data training sets to analytical scrutiny, such as the DHHS proposal mentioned above might require. Yet, some developers in the United States have balked at this suggestion of “fairness through transparency,” claiming that their models are proprietary and protected from public scrutiny [30,33,34]. Their pushback is largely prompted by their companies having heavily invested in research and development and fearing that their competitors will benefit from their cost outlays and be better positioned in the marketplace [35]. Thus far, courts and other regulatory agencies have refused to take a position on fairness through transparency, possibly at least owing to the immense lobbying pressures that large IT corporations can exert on state and federal legislators in protecting their business interests [36].

A fourth and very common recommendation for achieving fairness is by readjusting the model’s computational networks through re-weighting its sensitive or protected variables so that an historically oppressed group is treated “the same” as the majority or historically favored group. For example, one might evaluate the fairness of a model by changing the racial identity, age, sex, or residence location of members of the various sub-populations to see whether majority or historically advantaged group members would receive the same or a lesser level of benefits if they were counterfactually re-identified as minority persons [37].

This fourth approach to achieving fairness—which is sometimes labeled “fairness as demographic or statistical parity” [18]—stipulates that the likelihood of a positive outcome, like receiving a life-saving medication in times of acute medication scarcity, should be the same whether an individual is or isn’t in a protected group. It captures the essence of non-discrimination whereby equals—or people who are considered equals—are treated as equals [38].

The problem that fairness as demographic parity presents is when people do not present as equals in a morally obvious way, and moral disagreement arises about whether 1) they or their circumstances should be “equalized” and, if so, 2) what form that “equalizing” should take. Consider the following hypothetical scenario:

Case 1. A new and powerful strain of COVID has appeared. Hospitals once again find themselves without sufficient resources and begin developing ML models to help with triage-based care allocation computations. Early testing of the algorithms indicates they are easily able to distinguish patients who are economically better off and are usually in better pre-morbid health from ones who are poorly off. Not surprisingly, the models typically predict better outcomes and hence recommend more aggressive care for the former. Some model developers, however, argue that the models’ distributional weights should be adjusted to favor impoverished, especially homeless, patients. They believe that these populations have largely been victimized by historically unjust social structures and practices that explain their dire life circumstances, their heightened susceptibility to the virus, and their poorer prognosis. Other data scientists disagree, arguing that everyone is responsible for his or her station in life, and everyone should be treated similarly (or according to what they can pay for). Yet a third group of data scientists promotes an entirely different view: The algorithm, they argue, should promote social utility, not fairness. For them, the model’s allocational weights should be adjusted to favor essential workers like health professionals, food producers and distributors, transit workers, and sanitation personnel.

In this scenario, the first group argues that demographic parity should be achieved by, actually another, i.e., a fifth, definition of “fairness as distributive equity.” They believe it a social justice imperative to provide tangible benefits or advantages directly to groups who have historically suffered from unfair social practices and policies [15]. Those advantages would help to “equalize” their right to social goods as compared to the claims of majority group members. But they are opposed by another group who reject providing an outright benefit to the less well-off and instead favor a sixth notion of fairness: as “distributive equality” where everyone is treated the same [39]. Whereas the equity group might insist that a predictive model favorably weight a protected category like income or race—because they may token social inequity—the equality group might want to delete or de-emphasize such variables and simply have the model predict an outcome (and its associated treatment recommendation) on purely clinical criteria. The equality group can argue that the equity group advocates policies that are unfair to other socioeconomic groups and might repudiate any model that is weighted to favor the less well-off [40]. Notice, too, the third group problematically believes that fairness should not figure prominently in the algorithmic weighting affecting allocation as they favor a social utility approach.

An attempt to remediate the equity versus equality debate invites a seventh fairness characterization as “equal opportunity fairness.” Equal opportunity fairness, at least as it has developed in the United States, repudiates the practice of algorithmic models being re-calibrated to give certain job, credit card, or medical school applicants a direct or immediate algorithmic/mathematical “boost” that advantages them over others [39–41]. Indeed, United States courts, especially the U.S. Supreme Court, have ruled that certain direct and immediate advantaging practices, such as hiring quotas or a seat in a medical school class that favors members of an historically disadvantaged group, may be discriminatory to other competing groups [40–43]. Instead, this fairness approach focuses on barriers to opportunity, like sub-standard education, housing, or poor access to health care, and seeks to improve these health determinants so as to equalize “opportunity of access” for everyone. As such, this approach might not affect the moral decision making of model developers because algorithms do not directly engage in social justice efforts that seek to remediate various social determinants of health like income, race, education, etc. In any event, continuing public health research on health inequities suggests the ongoing failure of opportunity remediation on health disparities in the U.S., e.g., that disease rates for diabetes, hypertension, asthma, and stroke are worsening in Black populations as compared to whites [44].

How, then, might a morally conscientious ML development team and a clinician user group that is collaborating either in a research or business/purchasing capacity decide on all this, which suggests an eighth approach to characterizing fairness, namely, “fairness as the outcome of democratic processes” [45–47]. Perhaps the most popular strategy for achieving a democratic voice bearing on fairness in algorithmic development is to recruit culturally diverse teams that will periodically examine models for bias that then “enable the platform designers (and others) to identify ethical, and sometimes legal, issues and values that were not foreseen at the design stage, and to take additional measures to address these properly.” [46] But related problems immediately arise. First, even if development team members display some modicum of cultural diversity, there is no guarantee that they would bring an ethically sufficient range of ideological diversity to problems like those presented by the above. Furthermore, even if they did, we would still be without a way to respond to questions like what metrics should inform us as to when “diversity” has been achieved in a team, how one defines the essential properties of “culture” to ensure they are adequately represented among team members, and, most concerning, how to settle fairness disputes among team members.

What the above suggests—and its fairness characterizations and associated problems are summarized in Table 1—is that although pursuing a universal or one-size-fits-all definition of fairness is intellectually and pragmatically attractive, the elusive nature of that definition strongly suggests that fairness only makes sense in a particular context with a specific use or application. Indeed, the dense contextuality of fairness might partially explain the paucity of regulatory oversight in the United States, as well as why “the field lacks a design methodology to help AI design teams of members from different backgrounds brainstorm and surface potential fairness issues during the design stage.” [45, p.32].

**Table 1:** Fairness definitions mentioned in this paper and their barriers.

Fairness As	Typical Intervention	Barrier
Unawareness	Delete protected or sensitive label	Counterproductive if the sensitive variable is a strong predictor; model might infer protected label despite efforts to remove it
Equalized odds	Adjust odds ratios among sub-populations	Cannot be achieved if multiple fairness formulations are used and baseline rates differ among impacted sub-populations
Decisional transparency	Disclose model’s algorithm and training data	Intellectual property rights that are shielded by law from public scrutiny
Demographic/statistical parity	Adjust model’s weights to favor a certain sub-population so they might be treated similarly to everyone else	Allegations that a mathematically or algorithmically created advantage is “undeserved”
Distributional equity	Provide an advantage to an historically oppressed sub-population	Advantaging one population can disadvantage another
Distributional equality	Ensure all sub-populations receive an equal or the same share	Leaves open the question of what a “share” means and can be insensitive to different levels of neediness that distinguish sub-populations
Equal Opportunity	Ensure all sub-populations have reasonable if not equal access or opportunity to a social good without guaranteeing reception of the good	Requires political will and a government’s fiscal commitment to create access, which might not exist
Democratic Process	Ensure decision makers represent all individuals impacted by the model	Selecting criteria to decide the membership and ideological spread of a “democratic” constituency

Nevertheless and despite this dizzying array of fairness manifestations, no one in the model developer to user chain of operation wants to deploy a blatantly unfair model, at least because it could cause immense harm through misdiagnoses, inappropriate treatment recommendations, or civil rights violations like denial of benefits or entitlements. In addition to the harm any of these adversities might cause, they can also expose developers and clinicians to lawsuits, federal investigations, and along the way, considerable reputational loss [48].

Consequently, although it appears reasonable to task model developers with creating fair models—since they collect and curate the data, develop the model’s computational system, etc.—we endorse Xivuri’s and Twinomurinzi’s recent work on “the need to involve the society affected by the model in the development process and establish a platform for all AI stakeholders to participate.” [49] Indeed, the collaborations they envision go beyond just a developer-clinician collaboration and require “bring[ing] customer collaboration into the design development and deployment of responsible AI. Engage customer advisory councils, drawn from a broad cross-section of our customer base in the product development lifecycle to gain feedback around our development themes related to AI and ML.” [49]

Part II of this essay discusses how various moments in these collaborations might proceed, especially in instances where model developers, clinician users, and the lay public might disagree over a model’s fairness and, hence, need to create or negotiate justifiable conceptions of fairness that inform the model’s computations.

## Part II

### Moral character and fairness in ML development

The above suggests that an absence of regulatory guidance that might provide reliable moral co-ordinates for fairness analyses combined with a bewildering array of fairness definitions or characterizations explain why fairness might figure as an afterthought. That afterthought status can be present not only among professional ethics groups but among model developers themselves, whose data scientists typically hail from engineering training that has paid little attention to it [50]. As noted above, however, no model developer desires to create an obviously unfair model and as we discuss below, many model developers will go to considerable lengths to ensure that their models are fair. Indeed, some model developers have been keenly concerned about algorithmic fairness for years and have developed a host of bias mitigation tools to improve it such as IBM’s AI Fairness 360, Microsoft’s open-source toolkit Fairlearn, the University of Chicago’s Aequitas, Google’s TCAV, or Oracle’s Skater [51,52].

Despite these fairness interventions, however, clinician users might nevertheless wonder how committed model developers are to algorithmic fairness and social justice. Kate Kaye, for example, has noted that model developers occasionally use fairness tools “at the end of a development process, as a last box to check before a product or machine-learning model is shipped.” [53] The moral ideal, of course, would be to contemplate and analyze biasing traps or pitfalls at the inception of a project and then maintain that critical posture throughout the model’s development and deployment. But as Tricia Griffin and her colleagues recently reported on their study of moral agency in data scientists, one of her interviewees noted that “we’re not wondering if what we’re doing is ethical or not” [50]. That experience could then be compounded by the way many data scientists working on a model can feel morally disengaged from its intentions and impact, especially if management appears disinterested about fairness in the model’s project design [54]. Of course, a third factor that might dilute fairness considerations in model development is that model developers often work for companies that are largely motivated by profit maximization pressures that can subordinate fairness considerations to returns on investment. More than one of Griffin’s respondents noted how the promise of money, fame or “their personal brand” could and sometimes do obscure or taint workers’ ethical sensibilities [50].

A fourth factor, however, that calls for the kind of thoughtful collaboration on fairness that Xivuri and Twinomurinzi [49] encourage is perhaps the most interesting: When specific use cases present moral perplexities that invite *multiple stakeholder perspectives* on whether or not a model is fair or unfair. For example, consider the following three hypothetical cases:

Case 2. Patients who are scheduled for clinic appointments and then don’t appear can represent a significant revenue loss to an institution. At a meeting with clinical leadership, a hospital’s informatics group announces that it has been working with a commercial AI developer to create an ML model that predicts these “no-shows.” Once the model predicts a likely no-show, it will then double-book the slot so as to ensure that worker productivity (and related hospital income) is not lost. Although double-booking is not uncommon in the United States, some personnel at the clinic oppose the plan, pointing out that when the model is wrong, clinicians’ workloads will be stressed, patients’ wait times will increase, and their clinical visits will be (problematically) shortened. Clinicians note that this will especially and unfairly affect less well-off persons who are more likely to miss appointments for various reasons, often beyond their control. The model developer and the hospital’s executive leadership robustly disagree. They argue that if a certain segment of the hospital’s patient population is statistically likely to miss appointments, then they should bear whatever burdens result. Moreover, executive leadership argues that the hospital’s precarious financial position demands that all potential sources of revenue loss be mitigated as much as possible and that the financial survival of the hospital trumps the inconvenience of a longer wait time for certain patients. [55–57]

Case 3. A mortality prediction model has come into widespread use across the United States. Originally used to identify patients who need more intense or a frankly different program of care, ethically astute clinical personnel have begun to suspect that the model’s mortality predictions have become self-fulfilling prophecies. That is, once the model delivers a high probability prediction of an inpatient’s demise, some clinical personnel aggressively initiate conversations on discontinuing or withholding further intensive care measures with the patient or his or her family members in the hope of persuading them that such care would be “futile.” Some staff are deeply distressed by this, which they feel especially discriminates against the less well-off who oftentimes have meager, if any, health insurance. They argue that the model can reflect abundant historical bias: The life circumstances of less well-off persons dispose them to heightened illness acuity while their reduced financial circumstances might have historically prevented their accessing needed care. Furthermore, the medical records of less well-off persons are known to be particularly prone to inaccuracies, which can reduce a mortality prediction model’s accuracy. Nevertheless, other staff argue the model’s value in conserving revenues and reducing caregiver stress by preventing “futile” care allocations [58,59].

Case 4. A mental health facility has been approached by a generative AI developer whose model can considerably reduce the staff’s time spent in documentation. The model can generate patient summaries, clinical notes, treatment recommendations, and insurance billings as well as respond to patients’ questions and concerns. While the model is attractive to the facility’s business owners in terms of its relieving staff from these tasks and conserving revenues, some clinicians are fearful of confidentiality breaches. They argue that mental illness can be very stigmatizing and that the developer who owns the model will have access to extremely sensitive data. If that data is breached, released without warrant, or inadvertently placed on a public database, the psychological harms to the facility’s patients could be devastating. The facility’s shareholders and the clinical staff find themselves at an impasse over whether the model’s use should be adopted [60,61].

Cases like these illustrate the need for ML developers and at least their clinician users to exert as much moral insight and perspective as possible so as to identify relevant fairness/unfairness “flashpoints.” For example, should a clinic or hospital’s leadership even consider a double-booking algorithm since it will impose discomfort and burden on certain patient groups? Alternatively, if the clinical entity’s financial survival is truly at stake, developers and clinician users might collaborate on modifying the model. For example, the model might be reconfigured to double-book likely no shows who only require short visits or visits that might not require a physician’s attention [56]. Or the model might “examine” the clinic’s weekly workflow and double-book patients during predictably light periods or down-times. It might even build in a few minutes between all that day’s patient appointments to give clinicians a rest or some extra time that can be used to fully attend to patients who have been double-booked [57].

Another important factor in model developer-user collaborations is how model developers should be transparent as to how their datasets are created, curated, validated, implemented, and shared, as a failure to do this would taint any convincing justification that the model is “fair” [10,62–64]. Ideally, developers would exhibit heightened ethical awareness by producing algorithms whose “fair” outcomes are particularly sensitive towards minority groups or the sub-populations affected by the model [17]. They would be willing to explain and justify why a particular data set was used and what bias mitigation strategies were deployed and why. Developers and users might subscribe to policies like the United Kingdom’s Medicines and Healthcare Products Regulatory Agency’s stipulation: namely, that all entities selling products that leverage artificial intelligence (AI) demonstrate that their training and validation sets use data that are adequately representative of all patient populations in a user’s catchment area [11]. Or they might deploy quality assessment tools like the quality assessment of diagnostic accuracy studies (QUADAS-AI) to evaluate data sets for risk of bias [65].

In this regard, case study 3 reflects the challenge of inaccurate data, especially among socioeconomically marginalized populations. As an alternative, rather than use the model to urge the withholding or discontinuation of care straightaway, a model developer-clinical user collaboration might rethink the model to identify these (likely) terminally ill patients for more aggressive but compassionate end-of-life conversations [59]. The model might recommend and facilitate a referral to palliative care clinicians and even suggest to them certain talking points, relevant to the patient’s social, medical, or spiritual history, around which to build a compassionate end of life conversation.

Extending beyond the model developer-clinician user collaboration, the ethical characteristics of a commitment to fairness inevitably entail a willingness to engage in public conversations. For example, the AI Policy Forum that is convened by the MIT Schwarzman College of Computing brings together scientists, technologists, policymakers, and business leaders to discuss the societal challenges of artificial intelligence [66]. Similarly, The World Economic Forum has convened a Global Future Council on Artificial Intelligence whose participants identified practical interventions for companies to employ to assure AI Fairness [67]. And the United States FTC has held hearings on predictive analytics and guidelines for businesses using artificial intelligence [9,68]. Some commentators would like to see evaluations and reports resulting from meetings like these being directed to a newly created national office, perhaps like the FDA, which has a particular interest in human welfare especially concerning the less well-off [29]. Somewhat like the U.S. Centers for Disease Control and Prevention, that office might issue advice or directives to model developers and users on best practices.

These kinds of public conversations would be especially valuable in instances such as case study 4, which implicates serious confidentiality risks. Relevant questions, at least in the United States, might involve a public debate on whether or not patients should be informed that the confidentiality of their information cannot be guaranteed despite promises of anonymity [60,61]. A public airing of views in a case like this might provoke legislative action that would legally hold model developers as a “business associate” of the mental health facility. In American (HIPAA) law, a legally designated business associate is as obligated to protect the confidentiality of information which it receives as are the clinicians who source the material [69]. Furthermore, a clinician user group might only want to contract with model developers who can demonstrate robust and elaborate data security precautions.

In any case, the task of maintaining a sensitivity towards fairness will continue as long as ML models evolve and are deployed. New technology, new practice standards, and shifting healthcare demographics will persistently affect algorithmic accuracy and value over the lifetime of a model. Just as a one-size-fits-all fairness definition seems unlikely, a one-time, cross-sectional assessment and remediation of a model will not ensure the model’s fairness over its lifetime.

### Towards a lifetime ethics audit of ML models

A model satisfying certain technical and ethical criteria at its launch does not guarantee its long-term value. Commentators largely agree that evaluative processes should be in place for the life of the model for various reasons: inaccuracies due to bias or programming error might unexpectedly materialize (or the model doesn’t perform as intended); technologies and their operational environments might change; demographic patterns or patient populations might shift in ways that compromise accuracy and fairness; and never-ending system upgrades will require a continuing work relationship between model developers and users [29,68]. Indeed, after-market surveillance may enable increased trust in ML models in medicine, such as occurs in FDA long-term surveillance of drugs and medical devices in the U.S.

An element in a lifetime audit oversight intervention, for example, might be “transfer learning.” Transfer learning is a method that begins with an already trained model, say in image recognition, and enables a newer model to recognize original images that are re-produced via new imaging technologies [70]. The objective is essentially to repurpose an older model so as to keep pace with technological advances that require a newer one. Its key advantage is reducing costs because retraining a model from scratch can be prohibitively expensive. Nevertheless, there will come a time when a model reaches the end of its life, e.g., the training data are too outdated, model users require more complex computations, the physical infrastructure no longer supports the model, etc., and the model can no longer be transferred. Such moments are not without moral dimensions as clinician users will need to decide when the needs of their patients require the abandonment of older models.

The impact of machine learning models on patients introduces another consideration in the lifetime audit: the need for impact assessments. Such assessments would evaluate the model’s impact on users, its risks, the transparency and accessibility of its data sets, and its explainability and generalizability (and their limits) [29]. Ideally, risk assessment efforts would begin at the earliest stage of the model developer-clinician user relationship, as the above anticipates the distinct possibility of disagreement over whether a model’s design or outputs are fair or unfair. Thus, Krishna, et al underline the need for strategy and governance that include “principles, policies, and standards; roles and responsibilities; control processes and procedures” extending through all facets of the model development throughout its life cycle [71]. Important considerations that an impact assessment would include is a thoughtful analysis of the kinds of risk the model might pose to the end user; the degree to which senior management acknowledges and appreciates the presumptive risks involved; and a governance structure that adjudicates and manages the risks emanating from the model.

Importantly, impact assessments must include the lay public’s impression and assessment of the model’s purpose, the conditions and limits of its use, how its data are collected, curated, and shared, and how its deliverables are economically and ethically received [62,63,72]. However, this would initially require a significant effort to educate the public so that they be informed consumers and critical voices of ML models—a challenge that thus far remains sadly unattended [73].

All of these recommendations are ambitious, especially given the nascent stage of ML use in healthcare facilities, and all of them admit complex sub-tasks whose details will likely precipitate a host of valuative conflicts reflecting Western cultures’ moral pluralism. But to the extent that ML models may become standard of care technologies, they need to be vetted with an eye toward ethical uniformity bearing on fairness and will need to be monitored for unanticipated biases throughout their lifetime. Such an audit plausibly applies to any of the cases discussed above.

## Conclusion

We conclude by recalling observations by Milagros Miceli and her colleagues to “gaze upward” from the technical aspects of ML models and publicly investigate questions like: What are the goals of the corporate heads and investors of informational technology firms? What are the values that drive them? How strong and ethically informed are the voices working for them, especially individuals who create and test a model’s code? What are the hiring practices and preferences of AI developers and vendors? Do they evince sensitivity to issues of social inequities and in what ways? Can public dialogue be created around the inevitable biases that affect algorithmic models and is it possible to move towards conceptions of fairness embedded in that code that attract considerable consensus? Indeed, what kinds of AI savvy should candidates for public office display in their campaigns and, if elected, deliver on [11]?

ML models will not solve health disparities or their underlying inequities [41] but, like the FTC’s hearings, they might expose them and help mitigate them. However those efforts play out, the best outcome would foster those moral coordinates that advance global welfare, which is the ethical aspiration of the first articulation of AI decades ago [16]. The success or failure of that project will define the moral consciousness of this century’s research and development of ML models and its moral management of the ethically complex challenge that fairness presents. These efforts cannot be relegated to “afterthoughts” but need to be treated as critically pressing moral considerations affecting everyone impacted by ML technologies.

## References

[1] Gichoya JW, McCoy LG, Celi LA, Ghassemi M. Equity in essence: a call for operationalizing fairness in machine learning for healthcare. BMJ Health & Care Informatics. 2021;28:e100289. Available from: https://informatics.bmj.com/content/bmjhci/28/1/e100289.full.pdf. 10.1136/bmjhci-2020-100289 PMC8733939 33910923

[2] Google. (2018). Artificial intelligence at Google: Our principles. January 24, 2019. https://ai.google/responsibility/principles/.

[3] Future of Life Institute. (2017). Asilomar AI principles. October 23, 2018. https://futureoflife.org/open-letter/ai-principles/.

[4] Cruz RS, Liu X, Chan A-W, Denniston AK, Calvert MJ, the SPIRIT-AI and CONSORT-AI Working Group, et al. Guidelines for clinical trial protocols for intervention involving artificial intelligence: the SPIRIT_AI extension. Nat Med 2020;26:1351–63. 32908284 10.1038/s41591-020-1037-7 PMC7598944

[5] Corporation Microsoft. (2019). Microsoft AI principles. Retrieved February 01, 2019. https://www.microsoft.com/en-us/ai/our-approach-to-ai.

[6] Munn L. The uselessness of AI ethics. AI and Ethics. 2022. Available from: doi: 10.1007/s43681-022-00209-w

[7] Sanyal S. AI, machine learning, and big data: laws and regulations. Analytics Insight. 2021. Available from: https://www.analyticsinsight.net/ai-machine-learning-and-big-data-laws-and-regulations/.

[8] Kachra A-J, Hilliard A, Gulley A, Wilson I. Lawsuits in the United States point to need for AI risk management systems. OECD.AI. 2023. https://oecd.ai/en/wonk/lawsuits-usa-risk-management.

[9] Federal Trade Commission. FTC report warns about using artificial intelligence to combat online problems. 2022. https://www.ftc.gov/news-events/news/press-releases/2022/06/ftc-report-warns-about-using-artificial-intelligence-combat-online-problems.

[10] General Data Protection Regulation. No date. https://gdpr-info.eu/.

[11] Miceli M, Posada J, Yang T. Studying up machine learning data: Why talk about bias when we mean power? Proceedings of the ACM Human-Computer Interaction 6, GROUP. 2022. https://arxiv.org/pdf/2109.08131.pdf.

[12] No author. Beyond law: ethical culture and GDPR. Institute of Business Ethics. 2018. https://www.ibe.org.uk/resource/beyond-law-ethical-culture-and-gdpr.html.

[13] Mehrabi N, Morstatter F, Saxena N, Lerman K, Galstyan A. A survey on bias and fairness in machine learning. *arXiv*:*1908*.*09635v2 [cs*.*LG]*. 2019. https://arxiv.org/abs/1908.09635.

[14] Verma S, Rubin J. Fairness definitions explained. 2018 ACM/IEEE International Workshop on Software Fairness. 2018. https://fairware.cs.umass.edu/papers/Verma.pdf.

[15] Braverman P, Gottlieb L. The social determinants of health: it’s time to consider the causes of the causes. Public Health Reports. 2014;129:19–31. 10.1177/00333549141291S206 PMC3863696 24385661

[16] Dankwa-Mullan I, Scheufele EL, Matheny ME, Quintana Y, Chapman W, Jackson G, South B. A proposed framework on integrating health equity and racial justice into the artificial intelligence development lifecycle. Journal of Health Care for the Poor and Underserved. 2021; 32:300–317.

[17] Clark C, Wilkins CH, Rodriguez JA, Preininger AM, Harris J, DesAutels S, et al. Health care equity in the use of advanced analytics and artificial intelligence technologies in primary care. Journal of General Internal Medicine. 2021;36(10):3188–93. Available from: https://www.ncbi.nlm.nih.gov/pmc/articles/PMC8481410/. doi: 10.1007/s11606-021-06846-x 34027610 PMC8481410

[18] Lara MAR, Echeveste R, Ferrante E. Addressing fairness in artificial intelligence for medical imaging. Nature Communications. 2022;13:4581. Available from: doi: 10.1038/s41467-022-32186-3 35933408 PMC9357063

[19] Ho, D., & Xiang, A. (2020). Affirmative algorithms: the legal grounds for fairness as awareness. *The University of Chicago Law Review Online*. 2020. https://lawreviewblog.uchicago.edu/2020/10/30/aa-ho-xiang/.

[20] Ruf B, Detyniecki M. Active fairness instead of unawareness. 2020. arXiv:2009.06251v1[cs.AI]. 10.48550/arXiv.2009.06251.

[21] Centers for Medicare & Medicaid Services. Nondiscrimination in health programs and activities. Federal Register. 2022;87:47824–47920. https://www.regulations.gov/document/CMS_FRDOC_0001-3373.

[22] MacCarthy M. (2017). Standards of fairness for disparate impact assessment of big data algorithms. *Cumberland Law Review*. 2017;48:67–147.

[23] Chicco D, Jurman G. Survival prediction of patients with sepsis from age, sex and septic episode number alone. Nature Scientific Reports. 2020. Available from: https://www.nature.com/articles/s41598-020-73558-3. doi: 10.1038/s41598-020-73558-3 33051513 PMC7555553

[24] Cirillo D, Catuara-Solar S, Morey C, Guney E, Subirats L, Mellino S, Gigante A, Valencia A, Rementeria MJ, Chadha AS, Mavridis N. Sex and gender differences and biases in artificial intelligence for biomedicine and healthcare. 2020. *Npj Digital Medicine*. Available from: doi: 10.1038/s41746-020-0288-5 32529043 PMC7264169

[25] Dastin J. Amazon scraps secret AI recruiting tool that showed bias against women. *Reuters*. 2018. Available from: https://www.reuters.com/article/us-amazon-com-jobs-automation-insight/amazon-scraps-secret-ai-recruiting-tool-that-showed-bias-against-women-idUSKCN1MK08G.

[26] Etlinger S, Groopman J. The trust imperative: a framework for ethical data use. Altimeter. 2015. Available from: https://bigdata.fpf.org/wp-content/uploads/2015/11/Etlinger-The-Trust-Imperative.pdf.

[27] Angwin J, Larson J, Kirchner L, Mattu S. Machine bias. *ProPublica*. 2016. https://www.propublica.org/article/machine-bias-risk-assessments-in-criminal-sentencing.

[28] Paulus JK, Kent DM. Predictably unequal: understanding and addressing concerns that algorithmic clinical prediction may increase health disparities. Npj Digital Medicine. 2020:3:99; doi: 10.1038/s41746-020-0304-9 32821854 PMC7393367

[29] Ferryman K. Addressing health disparities in the Food and Drug Administration’s artificial intelligence and machine learning regulatory framework. Journal of the American Medical Informatics Association. 2020; 27(12):2016–2019. Available from https://www.ncbi.nlm.nih.gov/pmc/articles/PMC7727393/. doi: 10.1093/jamia/ocaa133 32951036 PMC7727393

[30] Xiang A. Reconciling legal and technical approaches to algorithmic bias. Tennessee Law Review. 2021;88(3):649–724.

[31] Karthik K. The impossibility theorem of machine fairness—a causal perspective. arXiv:2007.06024v1[cs.LG]. 2020. https://arxiv.org/pdf/2007.06024v1.pdf.

[32] Chouldechova A. Fair prediction with disparate impact: A study of bias in recidivism prediction instruments. 2016. *arXiv*:*1610*.*07524 [stat*.*AP]*. https://arxiv.org/pdf/1610.07524.pdf. 10.1089/big.2016.0047 28632438

[33] Bousquet C. Algorithmic fairness: tackling bias in city algorithms. *Harvard Kennedy School ASH Center for Democratic Governance and Innovation*. 2018. https://datasmart.hks.harvard.edu/news/article/algorithmic-fairness-tackling-bias-city-algorithms.

[34] New J. How to fix the Algorithmic Accountability Act. *Center for Data Innovation*. 2019. https://datainnovation.org/2019/09/how-to-fix-the-algorithmic-accountability-act/

[35] Burrell J. How the machine ‘thinks’: understanding opacity in machine learning algorithms. Big Data and Society. 2016:3:1. Available from: https://journals.sagepub.com/doi/epub/10.1177/2053951715622512.

[36] Birnbaum E. Tech spent big on lobbying last year. Politico 2022. Available at: https://www.politico.com/newsletters/morning-tech/2022/01/24/tech-spent-big-on-lobbying-last-year-00001144.

[37] Kusner M, Loftus J, Russell C, Silva R. Counterfactual fairness. 31st Conference on Neural Informaton Processing Systems. 2017. https://proceedings.neurips.cc/paper_files/paper/2017/file/a486cd07e4ac3d270571622f4f316ec5-Paper.pdf.

[38] Bloom P. People don’t want equality; they want fairness. The Atlantic. October 22, 2015. Available from: https://www.theatlantic.com/science/archive/2015/10/people-dont-actually-want-equality/411784/.

[39] Lamont J. Distributive justice. Stanford Encyclopedia of Philosophy. 2017. https://plato.stanford.edu/entries/justice-distributive/.

[40] Andre C, Velasquez M, Mazur T. Affirmative action: twenty-five years of controversy. Santa Clara University Issues in Ethics. 1992;5(2). https://www.scu.edu/mcae/publications/iie/v5n2/affirmative.html.

[41] Bent JR. Is algorithmic affirmative action legal? The Georgetown Law Review. 2020;108:803–853. Available from: https://www.law.georgetown.edu/georgetown-law-journal/wp-content/uploads/sites/26/2020/04/Is-Algorithmic-Affirmative-Action-Legal.pdf.

[42] Kim PT. Race-aware algorithms: Fairness, nondiscrimination and affirmative action. 2022. California Law Review. 2022;110:1539–1596.

[43] Gratz v. Bollinger, 539 U.S. 244 (2003). https://supreme.justia.com/cases/federal/us/539/244/.

[44] Heath S. Racial health disparities worsened over 20-year period. Patient Engagement HIT. 2020. https://patientengagementhit.com/news/racial-health-disparities-worsened-over-20-year-period#:~:text=November%2018%2C%202020%20%2D%20Racial%20health,better%20understand%20and%20mitigate%20disparities.].

[45] Panch T, Mattie H, Atun R. Artificial intelligence and algorithmic bias: Implications for health systems. *Journal of Global Health*. 2019. Available from: https://www.ncbi.nlm.nih.gov/pmc/articles/PMC6875681/pdf/jogh-09-020318.pdf. doi: 10.7189/jogh.09.020318 31788229 PMC6875681

[46] De Reuver M, van Wynsberghe A, Janssen M, Van de Poel I. Digital platforms and responsible innovation: Expanding value sensitive design to overcome ontological uncertainty. *Ethics and Information Technology*. 2020. Available from: doi: 10.1007/s10676-020-09537-z

[47] Zhang J, Shu Y, Yu H. Fairness in design: A framework for facilitating ethical artificial intelligence designs. *International Journal of Crowd Science*. 2023;7(1):32–39.

[48] Blackman R, Ammanath. Ethics and AI: 3 conversations companies need to have. Harvard Business Review. 2022. Available from: https://hbr.org/2022/03/ethics-and-ai-3-conversations-companies-need-to-be-having.

[49] Xivuri K, Twinomurinzi H. How AI deverlopers can assure algorithmic fairness. Discover Artificial Intelligence. 2023; 3:27. Available from: https://www.researchgate.net/publication/370176285_How_AI_Developers_Can_Assure_Algorithmic_Fairness#:~:text=Responsible%20organisations%20need%20to%20take%20deliberate%20actions%20to,testing%20for%20bias%20and%20corrective%20measures%20before%20deployment

[50] Griffin TA, Green BP, Welie JVM. The ethical agency of AI developers. AI and Ethics. 2023. https://www.semanticscholar.org/paper/The-ethical-agency-of-AI-developers-Griffin-Green/5fd53d4bdb7ec60e689f5a7c98c507a33d8acdde.

[51] No author. How to overcome AI bias…techniques and tools. Masaar Technology and Law Community. 2022. https://masaar.net/en/how-to-overcome-ai-bias-techniques-and-tools/.

[52] Gow G. How to use AI to eliminate bias. Forbes. 2022. https://www.forbes.com/sites/glenngow/2022/07/17/how-to-use-ai-to-eliminate-bias/?sh=730337e1f1fe.

[53] Kaye K. Why AI fairness tools might actually cause more problems. Protocol. 2022. Available from: https://www.protocol.com/enterprise/ai-fairness-tool-disparate-impact.

[54] Nichol A, Halley MC, Federico CA, Cho MK, Sankar PL. Not in my AI: Moral engagement and disengagement in health care AI development. Pacific Symposium on Biocomputing. 2023. Available from: https://psb.stanford.edu/psb-online/proceedings/psb23/nichol.pdf. PMC9782696 36541003

[55] No author. Rather than using overbooking or doublebooking to lessen the impact of patient no-shows, your practice could benefit from evaluating no-show trends. Professional Solutions. 2016. https://www.profsolutions.com/industries/physicians/insurance/risk-management/overbooking-and-double-booking-whats-acceptable/.

[56] Beltrami E, Grant-Kels JM. The doctor will see all of you now: Ethical dilemmas in double-booking patients. Journal of the American Academy of Dermatology. 2022 Dec 1:S0190-9622(22)03148-6. doi: 10.1016/j.jaad.2022.11.046 Epub ahead of print. . 36462632

[57] Duvefelt H. Double booking patients is difficult and destructive. 2016. KevinMD. https://www.kevinmd.com/2016/10/double-booking-patients-difficult-destructive.html.

[58] Choi MH, Kim D, Choi EJ, Jung YJ, Choi YJ, Cho JH, Jeong SH. Mortality prediction of patients in intensive care units using machine learning algorithms based on electronic health records. Nature Scientific Reports. 2022;12:7180, Available from: doi: 10.1038/s41598-022-11226-4 35505048 PMC9065110

[59] Lindvall C, Cassel C, Pantilat S, DeCamp M. Ethical consideraitons in the use of AI mortality predictions in the case of people with serious illness. Health Affairs Forefront. 2020. Available from: https://www.healthaffairs.org/content/forefront/ethical-considerations-use-ai-mortality-predictions-care-people-serious-illness.

[60] JAMA Kanter GP, Packel EA. Health care privacy risks of AI chatbots. JAMA. July 6, 2023. doi: 10.1001/jama.2023.9618 37410449

[61] Marks M, Haupt CE. AI chatbots, health privacy, and challenges to HIPAA compliance. JAMA. July 6, 2023. doi: 10.1001/jama.2023.9458 37410450

[62] Cuellar M-F, Huq A. The democratic regulation of artificial intelligence. Knight First Amendment Institute.2022. Available from: https://knightcolumbia.org/content/the-democratic-regulation-of-artificial-intelligence.

[63] Bishop L. Legal and ethical issues in curating big new data. GESIS and UK Data Service. 2017. https://dam.ukdataservice.ac.uk/media/604929/cessdaexpert_12-13sep17lb.pdf.

[64] No author. Benefits and harms of big data. The Center for Internet and Society. 2015. https://cis-india.org/internet-governance/blog/benefits-and-harms-of-big-data.

[65] Sounderajah V, Ashrafian H, Rose S, Shaw NH, Ghassemi M, Golub R, et al. A quality assessment tool for artificial intelligence-centered diagnostic test accuracy studies: QUADAS-AI. *Nat Med* 27, 1663–1665 (2021). Available from: doi: 10.1038/s41591-021-01517-0 34635854

[66] Massachusetts Institute of Technology. Q&A: Global challenges surrounding the deployment of AI. 2022. https://news.mit.edu/2022/qa-global-challenges-surrounding-deployment-ai-0926

[67] World Economic Forum. AI fairness is an economic and social imperative. Here’s how to address it. The Davos Agenda 2021. https://www.weforum.org/agenda/2021/01/how-to-address-artificial-intelligence-fairness/,

[68] Federal Trade Commission. Aiming for truth, fairness, and equity in your company’s use of AI. 2021. https://www.ftc.gov/business-guidance/blog/2021/04/aiming-truth-fairness-equity-your-companys-use-ai.

[69] U.S. Department of Health and Human Services Office for Civil Rights. Permitted uses and disclosures: Exchange for health care operations. 45 Code of Federal Regulations (CFR) 164.506(c)(4). 2016. https://www.hhs.gov/sites/default/files/exchange_health_care_ops.pdf.

[70] Diaz VF. The lifetime of a machine learning model. Towards Data Science. 2023. Available from: https://medium.com/towards-data-science/the-lifetime-of-a-machine-learning-model-392e1fadf84a.

[71] Krishna D, Albinson N, Chu Y. Managing algorithmic risks: Safeguarding the use of complex algorithms and machine learning. No date. Deloitte. https://www2.deloitte.com/content/dam/Deloitte/lu/Documents/risk/lu-risk-algorithmic-machine-learning-risk-management.pdf.

[72] Nagendran M, Chen Y, Lovejoy C, Gordon AC, Komorowski M, Harvey H, et al. Artificial intelligence versus clinicians: systematic review of design, reporting standards, and claims of deep learning studies. BMJ 2020;368:m689. Available from: https://www.bmj.com/content/bmj/368/bmj.m689.full.pdf. doi: 10.1136/bmj.m689 32213531 PMC7190037

[73] Kennedy B, Tyson A, Saks E. Public awareness of artificial intelligence in everyday activities. Pew Research Center. 2023. Available from: https://www.pewresearch.org/science/2023/02/15/public-awareness-of-artificial-intelligence-in-everyday-activities/.

